# Effect of Hyperthermia on Prothrombin Time (PT), International Normalized Ratio (INR), and Activated Partial Thromboplastin Time (APTT)

**DOI:** 10.7759/cureus.103566

**Published:** 2026-02-13

**Authors:** Jyoti Devi, Naveen Kakkar, Anuj Sharma

**Affiliations:** 1 Medical Pathology, Maharishi Markandeshwar Medical College and Hospital, Solan, IND; 2 Pathology, Maharishi Markandeshwar Medical College and Hospital, Solan, IND

**Keywords:** aptt, clotting times, hyperthermia, inr, pt

## Abstract

Background

Coagulation testing in the laboratory is performed at 37°C. However, this in vitro testing may not reflect the in vivo pathophysiology affecting coagulation factors in clinical conditions that cause hyperthermia. This study aimed to evaluate the effect of hyperthermia on prothrombin time (PT), international normalized ratio (INR), and activated partial thromboplastin time (APTT).

Methodology

This cross-sectional analytical study was conducted in a teaching hospital in North India over a period of one and a half years. In total, 50 patients aged >18 years were randomly selected from the samples received for coagulation testing in the laboratory. PT and APTT were determined by the standard manual tilt tube method within four hours of collection. For PT, uniplastin 5 (ISI-1.1), and for APTT, Liquicelin-E (Tulip diagnostics) was used. PT and APTT were determined at three different hyperthermic temperatures (39°C, 41°C, and 43°C). Testing at the standard 37°C was also performed. Parametric data were reported as mean and standard deviation, while non-parametric data were reported as median and interquartile range. The variation in PT/INR and APTT results at four designated temperatures was analyzed by repeated-measures analysis of variance. Statistical analysis was performed using SPSS version 16.0 (SPSS Inc., Chicago, IL, USA).

Results

Mean PT at 39°C, 41°C, and 43°C was 16.0 ± 7.1 seconds, 17.2 ± 9.6 seconds, and 19.7 ± 14.4 seconds, respectively. Mean PT at 37°C was 15.1 ± 4.9 seconds. The difference was statistically significant (p = 0.001). Mean INR at 37°C, 39°C, 41°C, and 43°C was 1.2 ± 0.4, 1.3 ± 0.6, 1.4 ± 0.9, and 1.6 ±1.4, respectively. The rising trend with increasing temperatures was significant (p = 0.004). Mean APTT at 37°C, 39°C, 41°C, and 43°C was 30.3 ± 11.6 seconds, 32.6 ± 11.5 seconds, 36.2 ± 13.2 seconds, and 42.5 ± 14.7 seconds, respectively. The difference was statistically significant (p < 0.001).

Conclusions

This study showed significant prolongation of PT, INR, and APTT at hyperthermic temperatures. The degree of prolongation of clotting times was greater in patients on oral anticoagulant therapy and those with liver disease.

## Introduction

In the hematology laboratory, routine coagulation testing is performed at 37°C. Results of coagulation testing are affected by many pre-analytical variables, which can adversely affect the accuracy of test results. These include the order of draw, altered blood to anticoagulant ratio (due to under- or overfilled sample containers), sampling technique, hematocrit, transport condition, storage temperature, centrifugation time, and reagent used [1-3]. As the in vitro coagulation testing is always performed at 37°C, it may not capture the potential abnormal in vivo functionality of coagulation factors in patients who are hyperthermic or hypothermic. There are many clinical scenarios where patients experience extremes of temperature. Hyperthermia occurs in heat stroke, certain drug reactions, acute febrile illness, extensive inflammation, and intense physical activity [4-7].

The process of coagulation involves a complex interaction between many different enzymatic proteins, platelets, and endothelium. The prolongation increases with rising temperature. Heat damages platelets and impairs their function, including adhesion, aggregation, and granule release. In severe hyperthermia, thrombocytopenia can occur. There is often an initial phase of suppressed fibrinolysis followed by enhanced fibrinolysis, which contributes to the complex coagulopathy seen in heatstroke [8]. Studies have shown that hyperthermia (temperature >37°C) prolongs the commonly ordered coagulation tests, namely, prothrombin time (PT), activated partial thromboplastin time (APTT), and international normalized ratio (INR) [9-11]. Hence, this study aimed to evaluate the effect of hyperthermic conditions on PT, APTT, and INR.

## Materials and methods

This cross-sectional analytical study was conducted in the Department of Pathology of a teaching hospital in Himachal Pradesh over an 18-month period (October 2023 to March 2025).

Sample size and selection of subjects

In total, 50 adult patients (>18 years of age) were included in the study. Sample size was estimated for the percentage frequency in a population with a design effect of 1.0. Based on a previous study [10] that showed significant coagulation derangement in 4.7% of hyperthermic patients, and a confidence interval of 90%, a sample size of 49 was obtained. Cases were randomly selected from samples received for PT and APTT in the hematology laboratory. Randomization was performed using the freely accessible website: Openepi (https://www.openepi.com/Menu/OE_Menu.htm). Cases with overfilled or underfilled containers, clotted, hemolyzed, and samples exceeding four hours of collection were excluded.

Sample collection and processing

Samples were collected in blue-top evacuated containers with 3.2% sodium citrate using 22-G or 21-G needles. Blood to anticoagulant ratio of 9:1 was maintained, and samples were processed within four hours of collection. Blood samples were centrifuged for 20 minutes at 2,000 g to separate the plasma. PT and APTT were estimated using a standard manual tube tilt method. PT and APTT were determined at three hyperthermic temperatures [39°C, 41°C, and 43°C], as well as at 37°C. The reagents and plasma were kept at the same temperature while testing in duplicate. Quality control was maintained by daily testing of pooled normal plasma.

A digital temperature-controlled and calibrated water bath was used for testing PT and APTT at different temperatures. From the set temperature, the new set temperature was attained in three minutes, thus not compromising the waiting time before testing. The base setting was kept at 37°C (tolerance limits: 37°C ± 0.5°C).

Reagents

For PT, Unipalstin 5 (ISI-1.1), and for APTT, Liquicelin-E (Tulip Diagnostics) was used to estimate the clotting times. All reagents were stored in a temperature-monitored refrigerator at 2°C-8°C.

Statistical analysis

For descriptive statistics, data were reported as mean and standard deviation. PT/INR and APTT result comparison for different hyperthermic temperatures was performed using repeated-measures analysis of variance (ANOVA). As Mauchly’s test indicated that the assumption of sphericity was violated for specialty, χ²(df) = X, p < 0.05; the Greenhouse-Geisser-corrected results are reported. Post hoc analysis with Bonferroni adjustment was also performed when the repeated-measures ANOVA was significant. Statistical analysis. including calculation of confidence intervals (CIs), was done using SPSS version 16.0 (SPSS Inc., Chicago, IL, USA). A p-value <0.05 was considered significant.

Ethical considerations

Consent was obtained from all patients. The study was approved by the Institutional Ethics and Research Committee, Maharishi Markandeshwar Medical College and Hospital (approval number: 23(2)-44).

## Results

In total, 50 patients were included in the study. Their age ranged between 18 and 80 years, with a mean age of 43 ± 15.7 years. The study included 28 males and 22 females, with a male-to-female ratio of 1.3:1. The clinical presentation of the 50 patients included in the study was varied (Table 1).

**Table 1:** Clinical features of patients included in the study. *: Acute febrile illness (2), hypothyroidism (1), conjunctival hemorrhage (1), and ocular trauma (1).

Clinical features	Number	Percentage
Chest pain	9	18%
Routine checkup	9	18%
Joint pains	7	14%
Antenatal checkup	7	14%
Cholecystitis	6	12%
Chronic liver diseases	4	8%
On oral anticoagulants for valvular heart disease	3	6%
Miscellaneous*	5	10%

PT, INR, and APTT at hyperthermic temperatures

**Figure 1:**
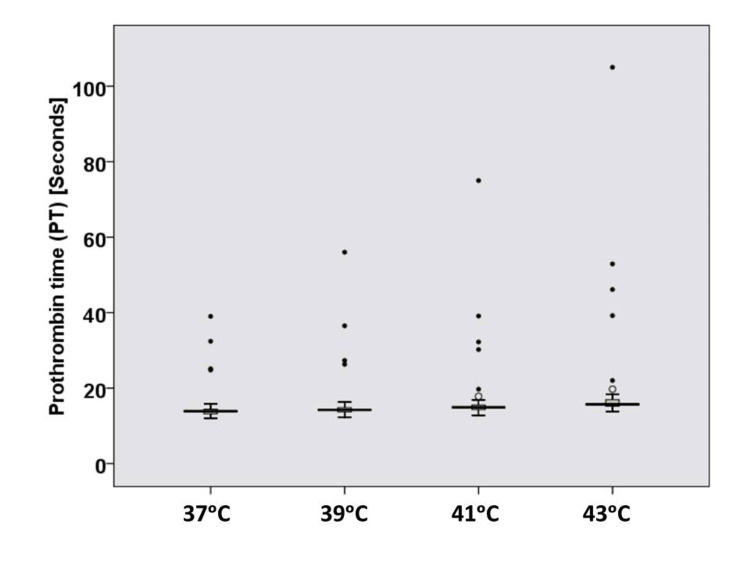
Box plot for prothrombin time results at hyperthermic temperatures (n = 50).

**Figure 2:**
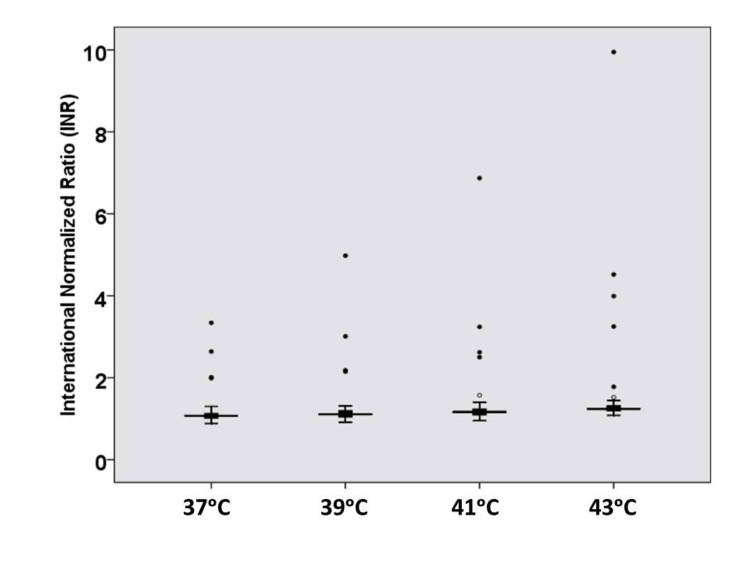
Box plot for international normalized ratio results at hyperthermic temperatures (n = 50).

The mean PT at hyperthermic temperatures (39°C, 41°C, and 43°C) and at 37°C is shown in Table 2 and Figure 1. The difference was statistically significant (p = 0.001). The mean INR values at hyperthermic temperatures (39°C, 41°C, and 43°C) and at 37°C are shown in Table 3 and Figure 2. The difference was statistically significant (p = 0.004).

**Table 2:** Prothrombin time results at hyperthermic temperatures and at 37°C. SD: standard deviation; CI: confidence interval

Parameter	37°C	39°C	41°C	43°C	P-value
Range (seconds)	12–39	12.3–56	12.8–75	13.8–105	0.001
Mean ± SD (seconds)	15.1 ± 4.9	16.0 ± 7.1	17.2 ± 9.6	19.7 ± 14.4	
95% CI	13.7 to 16.5	13.3 to18.7	14.5 to 19.9	15.7 to 23.7	

**Table 3:** International normalized ratio results at three hyperthermic temperatures and at 37°C. SD: standard deviation; CI: confidence interval

Parameter	37°C	39°C	41°C	43°C	P-value
Range (seconds)	0.88–3.34	0.91–4.98	0.95–6.87	1.08–9.95	0.004
Mean ± SD (seconds)	1.2 ± 0.4	1.3 ± 0.6	1.4 ± 0.9	1.6 ±1.4	
95% CI	1.1 to 1.3	1.1 to 1.5	1.2 to 1.6	1.2 to 2.0	

Of the 50 patients included in the study, in four patients, the INR values were >2.0 at hyperthermic temperatures. Three of these patients were on oral anticoagulant therapy for cardiac indications, while the other patient had jaundice due to chronic liver disease. All four patients also had prolonged APTT values. Three other patients had normal PT/INR and prolonged APTT values at hyperthermic temperatures. Two of these were on anticoagulants, while one patient had hypothyroidism.

**Figure 3:**
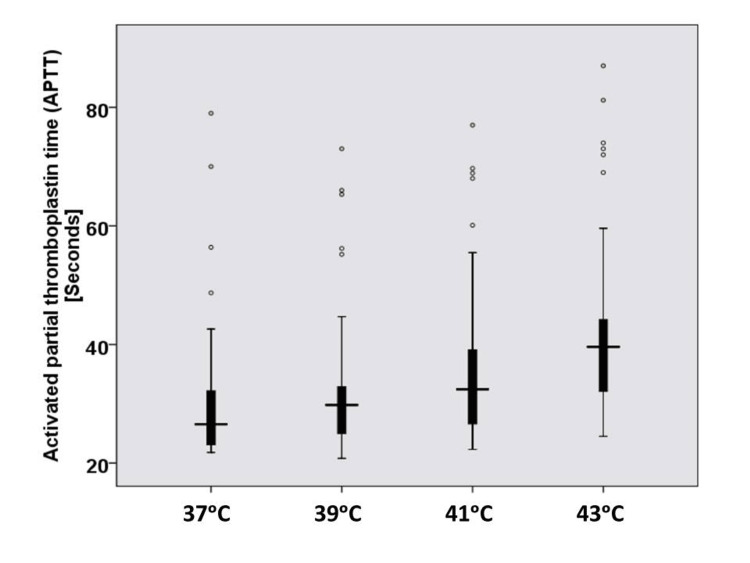
Box plot for activated partial thromboplastin time results at hyperthermic temperatures (n = 50).

The mean APTT values at hyperthermic temperatures (39°C, 41°C, and 43°C) and at 37°C are shown in Table 4 and Figure 3. The difference was statistically significant (p < 0.001).

**Table 4:** Inter-temperature comparison of mean PT, INR, and APTT. PT: prothrombin time; INR: international normalized ratio; APTT: activated partial thromboplastin time; SD: standard deviation; CI: confidence interval

	PT	INR	APTT
Temperatures	Mean ± SD	Mean ± SD	Mean ± SD
	(seconds)	(seconds)	(seconds)
37°C	15.1 ± 4.9	1.2 ± 0.4	30.3 ± 11.6
39°C	16.0 ± 7.1	1.3 ± 0.6	32.6 ± 11.5
P-value	0.068	0.083	0.002
37°C	15.1 ± 4.9	1.2 ± 0.4	30.3 ± 11.6
41°C	17.2 ± 9.6	1.4 ± 0.9	36.2 ± 13.2
P-value	0.027	0.054	<0.001
37°C	15.1 ± 4.9	1.2 ± 0.4	30.3 ± 11.6
43°C	19.7 ± 14.4	1.6 ± 1.4	42.5 ± 14.7
P-value	0.01	0.023	<0.001
39°C	16.0 ± 7.1	1.3 ± 0.6	32.6 ± 11.5
41°C	17.2 ± 9.6	1.4 ± 0.9	36.2 ± 13.2
P-value	0.016	0.051	<0.001
39°C	16.0 ± 7.1	1.3 ± 0.6	32.6 ± 11.5
43°C	19.7 ± 14.4	1.6 ± 1.4	42.5 ± 14.7
P-value	0.007	0.019	<0.001
41°C	17.2 ± 9.6	1.4 ± 0.9	36.2 ± 13.2
43°C	19.7 ± 14.4	1.6 ± 1.4	42.5 ± 14.7
P-value	0.006	0.014	<0.001

Inter-temperature comparison

Inter-temperature comparison for PT at 37°C and 39°C showed no significant difference (p = 0.068). Inter-temperature comparison for PT for all other temperature sets showed a significant difference (p < 0.05). Inter-temperature INR comparison between 37°C and 39°C, 37°C and 41°C, and 39°C and 41°C showed no significant difference (p > 0.05). Inter-temperature INR comparison for all other temperature sets showed a significant difference (p < 0.05). Inter-temperature APTT comparison for all temperature sets showed a significant difference (p < 0.05) (Table 5).

**Table 5:** Activated partial thromboplastin time results at three hyperthermic temperatures and at 37°C. SD: standard deviation; CI: confidence interval

Parameter	37°C	39°C	41°C	43°C	P-value
Range (seconds)	21.8–79	20.8–73	22.3–77	24.5–87	<0.001
Mean ± SD (seconds)	30.3 ± 11.6	32.6 ± 11.5	36.2 ± 13.2	42.5 ± 14.7	
95% CI	27.1 to 33.5	29.4 to 35.8	32.5 to 39.9	38.4 to 46.6	

## Discussion

This study showed significant prolongation of PT, INR, and APTT at hyperthermic temperatures when mean values were compared. Inter-temperature comparison for all chosen temperatures also showed significant differences for PT and INR, except for a few temperature sets. APTT showed significant prolongation for all inter-temperature comparisons.

A study analyzed hemostatic parameters in 132 patients with heat stroke. Compared to the control group, PT and APTT prolongation was seen in patients with heat stroke (p < 0.05) [9]. In a retrospective study of 171 patients with peritoneal carcinomatosis, the authors studied coagulation changes after cytoreductive surgery (CRS) and hyperthermic intraperitoneal chemotherapy (HIPEC). Among other parameters, INR and APTT were also studied. Abnormal INR and APTT were defined as INR >1.5 or APTT >45 seconds. Severe coagulation derangement was defined as INR >3.0 and APTT >60 seconds. When median preoperative and postoperative results were compared, a significant difference (p = 0.007) was seen for INR. APTT did not show a significant difference (p > 0.05). Coagulopathy was seen in 38% patients and severe changes in 4.7% patients [10].

A study from the Netherlands evaluated the effect of different temperatures (range 28°-40°C) on PT and INR determined by two different thromboplastins: rTF/16 (human, recombinant) and RBT/16 (rabbit brain). The authors reported short PTs with rTF/16 when the reaction temperature was increased up to 39°C-40°C. PTs with RBT/16 were prolonged at higher temperatures [11].

Another study compared APTT and D-dimer values among 12 healthy individuals, 20 patients with classic heat stroke, and 13 patients with exertional heat stroke. APTT in the heat stroke group was significantly prolonged compared to the control group (p < 0.05) [12].

A study from China enrolled 302 patients with heat-related illnesses across 24 hospitals. Of these, 131 patients had heat stroke, including 36 patients with heat-induced coagulopathy. Mean PT in patients with heat stroke (core temperature 39.7°C) was 13.9 seconds and was significantly higher than that seen in the non-heat stroke (core temperature 38°C) group (p < 0.001). Mean INR was also significantly higher (1.2 vs. 1.0) (p < 0.001). Mean APTT in the heat stroke group was 31.2 seconds versus 29.8 seconds in the non-heat stroke group. The difference was statistically significant (p = 0.031) [13].

In contrast to our findings, a study conducted among eight healthy male volunteers evaluated the effects of a 45-minute thermoneutral bath followed by a 50-minute bath with increasing temperature of water until a maximum of 41°C. At a body core temperature of 39°C, various coagulation parameters were studied. Hyperthermia showed shortening of APTT (p < 0.05) [14]. This could be explained as in our in vitro study, temperature control was extremely precise, while in this study, with water immersion, there could be a core body temperature mismatch with the set temperature.

A study evaluated APTT in vitro at different temperatures, i.e., 33°C, 35°C, 37°C, 39°C, and 41°C in normal plasma in response to unfractionated heparin. At test temperatures of 33°C and 41°C, heparin-induced prolongation of APTT was increased. APTT was prolonged significantly (p < 0.05) by 22% at 41°C compared with APTT at normothermic test temperature [15].

In clinical practice, the effect of coagulation derangements in hyperthermia has an adverse effect on patient outcomes. In a multicentric study in patients with heat stroke, a routine coagulation screen was performed within 24 hours of presentation. In this study, the 60-day mortality was 20.9%. Median PT, INR, and APTT of non-surviving patients were significantly higher than those of the survivors (p < 0.01) [16].

A study analyzed data from 176 patients with severe heat stroke. Based on the outcome, they were divided into the survival group (n = 150) and the non-survival group (n = 26). PT and APTT assessed within 24 hours of admission were analyzed. PT showed significant prolongation in the non-survival group (PT = 34.0 and 18.4 seconds, respectively). APTT also showed a similar pattern with a mean APTT of 79.7 seconds in the non-survival group versus 60.8 seconds in the survival group (p = 0.000) [17].

Previous studies and findings of our study have shown significant coagulation derangements at hyperthermic temperatures. It is, however, unlikely that laboratories will customize coagulation testing for these rare situations. However, in the clinical setting of significant hyperthermia, when coagulation results are discordant with the clinical profile, temperature-specific laboratory testing may be considered. Coagulometer programs could also have options for altering incubation temperatures other than the pre-set 37°C.

Our study was performed with one reagent set for PT and APTT. Further studies with different reagents can highlight the effect of reagent variability in hyperthermic conditions. The majority of the patients in our study showed normal PT/INR and APTT values at 37°C, which showed prolongation at higher temperatures. The representation of patients with baseline prolonged clotting times at 37°C in our study was low. In patients who were on oral anticoagulants or had liver disease, the prolongation of clotting times at higher temperatures was clinically significant (INR >2.0).

## Conclusions

This study showed significant prolongation of PT, INR, and APTT at hyperthermic temperatures. The maximum derangement was seen at the highest tested temperature of 43°C for PT, INR, and APTT. The degree of prolongation of clotting times was greater in patients on oral anticoagulant therapy and those with liver disease. In these patients, the prolongation was clinically significant.
